# AML-derived Extracellular Vesicles Confer *De Novo *Chemoresistance to Leukemic Myeloblast Cells by Promoting Drug Export Genes Expression and ROS Inhibition

**DOI:** 10.22037/ijpr.2020.113272.14199

**Published:** 2021

**Authors:** Mohieddin Barzegar, Mehdi Allahbakhshian Farsan, Vahid Amiri, Saeed Mohammadi, Shaghayegh Shahsavan, Amin Mirzaeian, Mohammad Hossein Mohammadi

**Affiliations:** a Department of Hematology and Blood Banking, School of Allied Medical Sciences, Shahid Beheshti University of Medical Sciences, Tehran, Iran.; b HSCT Research Center, Shahid Beheshti University of Medical Sciences, Tehran, Iran.; c Hematology, Oncology and Stem Cell Transplantation Research Center, Tehran University of Medical Sciences, Tehran, Iran.

**Keywords:** Multidrug resistance, Relapse, Extracellular vesicles, Acute myeloid leukemia, De novo

## Abstract

In spite of successful initial remission, chemo-resistance and relapse are still concerning points in acute myeloid leukemia (AML) treatment strategies. Multidrug resistance (MDR) appears to be the major contributor of chemo-resistance, arising in some sub-clones of cancers and could be developed in others. The aim of this study was to investigate the role of extracellular vesicles (EVs) derived from AML patients on the transmission of chemo-resistance phenotype. Ultracentrifugation was employed to isolate EVs from healthy controls, new cases, and relapsed AML patients. The EVs size, morphology, and immunophenotype were determined by dynamic light scattering, TEM, and flow cytometry respectively. Bradford assay was performed to measure the protein content of EVs. MTT assay and flow cytometry analysis were also used to determine the viability index, induction of apoptosis, and ROS generation in U937 cells. The expression level of two efflux pumps was assessed using qRT-PCR analysis. Findings of TEM, DLS, and flow cytometry confirmed that EVs had a desirable shape, size, and surface markers. EVs derived from both new cases and relapsed AML patients significantly reduced idarubicin-induced apoptosis in the U937 cells. The analysis of drug efflux pumps gens revealed that EVs over-express *MRD1* and *MRP1* in the target cells. These findings suggested a novel role of EVs in mediating the acquired chemo-resistance in AML patients by inducing the expression of the drug efflux pumps; however, further investigations will be required to elucidate other underlying mechanisms of resistance that are mediated by EVs.

## Introduction

Acute myeloid leukemia (AML) is the commonest acute leukemia in adults. Despite basic advances in the understanding of AML pathogenesis, the patient’s clinical situation is still dismal and their therapeutic outcome has not kept pace in parallel with molecular findings (1, 2). Although complete remission (CR) occurs in most patients using conventional chemotherapy, the majority of them eventually succumb to disease relapse as a result of the development of resistant colons (3). The advances in the molecular understanding of leukemia have currently introduced AML as a disease with subtly different clonal hematopoietic cells and a vast variety of heterogeneous stromal cells (4). It is now realized that in spite of the monoclonal entity of hematologic malignancies, a marked heterogeneity is evolved in a myriad type of these malignancies as a result of an accumulation of different genetic and epigenetic changes over a prolonged period of time (5). With the favor of this phenomenon, many sub-clones will be shaped with different phenotypes, such as chemo-resistance and metastatic (6). It is well-defined that reciprocal dialogs between leukemic cells and their surrounding environment could be contributed to the horizontal transfer of the drug resistance (7). Extracellular vesicles (EVs) are one of the most important communication tools for establishing a dialogue between leukemic cells and the bone marrow microenvironment (8).

EVs are bi-layer lipid membrane particles released by both normal and neoplastic cells that could deliver their cargos to recipient cells by a variety of mechanisms, including ligand/receptor signaling, phagocytosis, and/or endocytosis (9). The amount and the content of EVs can vary depending on the type and the condition of originated cells; however, some cargos are systematically enriched in EVs (10). A mounting preclinical and clinical evidence indicated that EVs are strongly associated with transmitting cancer phenotype from cell-to-cell (10, 11). Recently, a horizontal transfer of drug export molecules has been suggested as a plausible mechanism in this pathway (12). Drug efflux pumps located at the cell membrane have been proved to expel out the xenobiotics from the cells to maintain their intracellular concentration at a sub-lethal level. Multidrug resistance generally results from the expression of ATP-dependent efflux pumps, which could induce resistance to the therapy in any field of medicine (12-15). Resistance to Vinca Alkaloids, anthracyclines, actinomycin-D, and paclitaxel are the most striking examples in the field of cancer therapy. 

Among a variety of drug resistance molecules, the multi-drug resistance gene (MDR1), also known as P-glycoprotein 1 (Pgp), and multidrug resistance protein 1 (MRP-1) are contributed to the treatment failure of anthracyclines in AML patients (16, 17). In a study conducted by Bouvy, it has been reported that anthracycline-resistant strain of HL60 cell line displayed over-expressed MRP-1and transferred EVs which could give rise to the resistance in the anthracycline sensitive strain (12). Additionally, Torreggiani and colleagues showed that exosomes derived from doxorubicin-resistant osteosarcoma cells could be uptaken by secondary cells and induced a doxorubicin-resistant phenotype (18). Wang demonstrated that through dysregulation of Rab8B and Rab5 function, chemotherapeutic agents could stimulate the secretion and recycling of ABCB1-enriched EVs, which in turn could ultimately induce chemo-resistance phenotype (19). Given the high rate of disease relapse in AML patients and the frequency of drug-resistance in this malignancy, we aimed to evaluate the effect of EVs derived from new cases and relapses in AML patients on the induction of resistance against idarubicin (IDA) in the U937 cell line. Then, to dissect the molecular mechanism through which EVs could induce resistance in IDA-sensitive U937 cells, the expression levels of *MRP1* and *MDR1* were evaluated. 

## Experimental

*Study population*

EVs were obtained from peripheral blood of 11 patients with AML, including 7 new cases before treatment and 4 relapsed AML patients who were referred to Taleghani hospital (Tehran, Iran). Sequentially, 10 apparently healthy subjects were included in this study as a control group. Informed consent was obtained from all individuals and was included in the study. All patients were diagnosed with a history of non-M3-AML. Samples were collected in heparin tubes. The detailed demographic and biological characteristics of patients were presented in Table 1.

*Isolation of Extracellular vesicles*

The volume of the plasma samples was variable. At first, the plasma samples were diluted with a similar volume of Phosphate Buffered Saline (PBS) and were centrifuged for 15 min at 3000 *×g*. The collected supernatants were then centrifuged at 12,000 *×g* for 20 min. The ultimate supernatants were transferred into 5 mL ultracentrifuge tubes (Beckman model. L5-50-USA) and were ultra-centrifuged at 110,000 *×g* for 90 min (all centrifugation steps were performed at 4 °C). The supernatants were then discarded, the pellet containing EVs were dissolved in 500 µL PBS and transferred to the -80 °C for further analyses. 

*EVs surface CD markers detection*

EV-enriched and suspended pellets were analyzed by a attune NxT cytometer (Thermo Fisher Scientific-USA). The system was calibrated using standard microbeads with diameters of 0.3–0.1–3 μm (BD-USA) to define the precise size of particles, which shall be gated as EVs in flow cytometry (20). EVs were characterized by flow cytometry using fluorescein isothiocyanate (FITC) - or phycoerythrin (PE)-conjugated antibodies. In the first step, pan EVs’ markers CD9 and CD63 were used to confirm the entity of EVs, and then AML CD markers, such as CD13, CD33, CD34, and HLA DR were evaluated.

*TEM and DLS*

Fifty microliter EVs from AML patient and healthy subjects were put on a copper grid coated with 0.125% Formvar in chloroform. The grids were stained with 1% v/v uranyl acetate in ddH2O and the samples were examined immediately. A JEOL 1011 transmission electron microscope was used for imaging (JEOL, Tokyo, Japan). The size distribution of EVs was determined using DLS by a Malvern Nano ZS instrument (UK). Briefly, the volume of each sample (patients and cell line) was increased up to 1 mL by PBS, loaded into a quartz cuvette, and measured at 25 °C. Finally, the results were analyzed using the Zetasizer software (version 7.11).

*Bradford assay*

The determination was carried out according to the manufacturer’s instructions (Kiazist, Iran) and spectrophotometric quantification of the protein content was measured at 595 nm. Protein levels were first measured separately for all the samples, then the samples from each group (newly diagnosed patients, relapse patients, and control group) were pooled separately and used for subsequent tests.

*Drug*

Idarubicin hydrochloride (Sigma-Aldrich, Germany) is an anthracycline anti-leukemic drug, which is commonly used in combination with cytosine arabinoside as a first-line treatment of AML. To obtain a 1 mg/mL stock solution, this drug was dissolved in phosphate-buffered saline (PBS) and the stock was kept at −20 °C. Actinomycin D, a chemotherapy drug with the ability to inhibit transcription, was provided from Minatajhiz Aria Company (Tehran, Iran). This drug was dissolved in Dimethyl sulfoxide (DMSO) to obtain a 0.5 mg/mL stock solution and stored at −20 °C.

*Cell culture*

The human AML cell line U937 was obtained from the Pasteur Institute (Tehran, Iran) and cultured in suspension in RPMI medium supplemented with 2 mM L-glutamine, 10% FBS, 100 units/mL penicillin, and 100 μg/mL streptomycin in a humidified 5% CO2 at 37 ºC. For Idarubicin (IDA) treatment, a relevant amount of stock solution (0.5 mM in RPMI) of IDA was added to a culture medium to attain the concentrations of 0.05, 0.1, 0.2, 0.3, 0.4, and 0.5 μM. All drug treatments were carried out in three independent experiments and all assays were performed in triplicate.

*MTT assay*

U937 cells (5 × 103) were seeded into a 96-well plate in the presence of IDA. After 36 h, the MTT solution (5 mg/mL in PBS) was added to each well and incubated at 37 °C for the next 3 h. The percentage of the metabolic activity of the cells was evaluated by dividing the optical densitometry (OD) of a resulting formazan measured by an enzyme-linked immunosorbent assay (ELISA) reader in the drug-treated groups by the OD of the control group. 

*Evaluation of Apoptosis by Annexin V/Propidium Iodide (PI) Assay*

To explore the effect of IDA, IDA-plus-healthy-EVs, IDA-plus-nAML-EV, and IDA-plus-rAML-EV on the induction of apoptotic cell death, the cells were subjected to Annexin-V/PI staining analysis. Drugs-treated cells were collected after 36 h of treatment and suspended in 100 μL of the incubation buffer. Then, 2 μL annexin-V-Flous was added to each sample and incubated for 20 min in the dark. The intensity of fluorescence was measured by flow cytometry. 

*ROS assay*

To determine the effect of IDA on the production of intracellular reactive oxygen species (ROS) in U937 cells, we used a fluorogenic dye DCFH-DA, for measuring hydroxyl, peroxyl, and other ROS activity within the cell. After incubation with IDA, the cells were incubated with DCFH-DA at 37 °C for 30 min. Finally, fluorescence intensities of the samples were detected by a fluorescence spectrophotometer (Cary Eclipse, USA) with excitation at 485 nm and emission at 530 nm.

*RNA extraction, cDNA synthesis, and quantitative real-time PCR*

Total RNA from U937 cells, AML patients, and AML derived EVs were extracted by RiboEx (GeneAll®- South Korea), and RiboEx LS TM (GeneAll®) respectively, according to the Protocol. After confirming the quantity of each extracted RNA by Nanodrop instrument, the reverse transcription reaction was performed using the PrimeScript™ 1^st^ strand cDNA Synthesis Kit (Takara Cat. #RR037A, Japan) and the synthesized cDNA were kept at -20 °C until use. Next, to examine the effect of IDA-plus healthy-EVs, IDA-plus-nAML-EV, and IDA-plus-rAML-EV on the expression of *MRD1* and *MRP1* in U937 cells, control group, and patients samples, thawed cDNAs were subjected to quantitative real-time PCR (qRT-PCR). The fold change values were calculated based on 2−ΔΔCt relative expression formula. Selected primers are shown in Table 2

*Statistical analysis*

The results obtain from three independent tests and presented as mean ± SD. Statistical analyses were analyzed using GraphPad Software (GraphPad Prism version 7.00 for Windows,) and the significance of differences between experimental variables was determined by the use of a two-tailed student’s *t*-test and one-way variance analysis. A probability level of *P* < .05 was considered statistically significant.

## Results

*Evaluating the characteristics of isolated EVs by flow-cytometry, DLS, and electron microscopy imaging*

*Flowcytometry*

**Figure 1:**
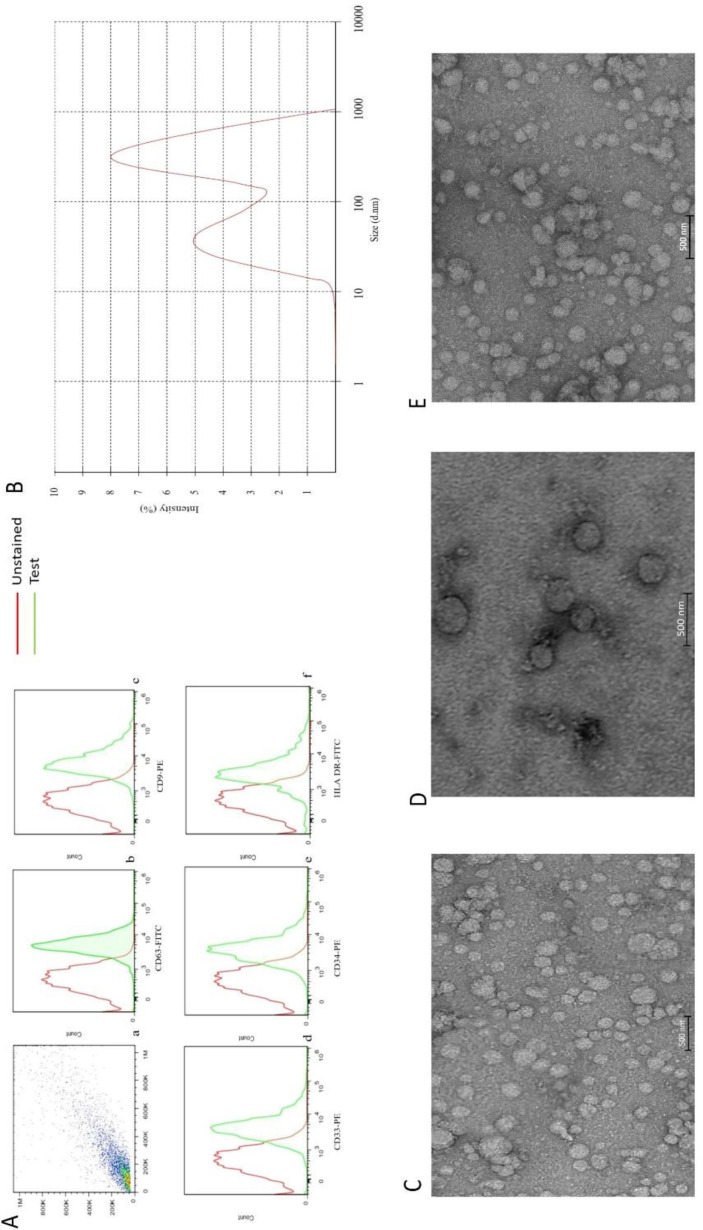
(A) a flow cytometry gate AML- EVs. Flow-cytometric analyses showed that the AML-EVs express CD63, CD9, CD33 CD34, and HLA DR (b, c, d, e, and f). (B) Measurement of the size range of EVs by DLS with the average size of 340 nm. (C, D, and E) TEM analyses of EVs, three groups of newly diagnosed patients, healthy subjects and relapsed patients, respectively

EVs isolated from the plasma of AML patients expressed CD34, HLA DR, and CD33, as well as CD9 and CD63. The presence of these markers confirmed that they were originated from myeloblasts. Although the expressional pattern of new cases and relapsed-relevant CD markers, CD9 and CD63 on EVs were similar, the expression of other markers on EVs was proportional to the percentage of blasts. While CD9, CD63, and CD33 were positive in the control group, the expression of CD34 and HLA DR were negative (data were not shown) (Figure 1).

*TEM and DLS analyze of EVs*

To investigate whether the structure of isolated EVs was completely conserved during the isolation process, a TEM technique was performed. As presented in Figure 1, the result showed that EVs had an intact structure. EVs size on electron microscopy was less than 1 µm. This data was also confirmed by DLS, which showed a curve with two peaks, one at 61.43 ± 14.12 nm and another at 589.5 ± 35.6. Moreover, these curves had a weighted intensity of 40.2% and 59.2%, respectively (Figure 1), indicating that the EVs were isolated properly.

*The comparison between EVs protein levels in healthy individuals, new cases, and relapsed patients*

**Figure 2:**
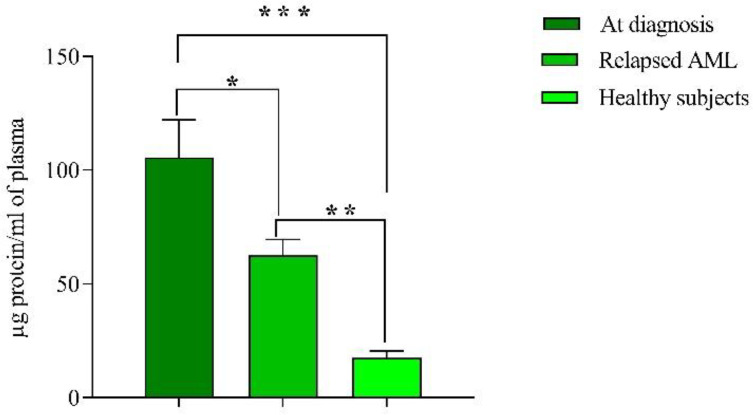
Mean protein content of EVs measured by Bradford method. Concentration of protein in new cases and relapsed groups were significantly higher than healthy subjects. Of note, the concentration of protein in new cases was also higher than relapsed cases. Data are mean ± SE of three independent experiments. Statistical significance was defined at^ *^P < 0.05, ^**^P < 0.01 and ^***^P < 0.001 compared to corresponding control

Mean protein content of EVs at diagnosis was 105.5 ± 16 µg/mL, which dropped to 62 ± 7 µg/mL in relapsed AML patients (*P* < 0.05). Ten apparently healthy subjects as controls were included in this study and plasma EVs were isolated and mean protein content was 17.5 ± 3 µg, which was significantly lower than both groups (at diagnosis and relapsed patients), (*P* < 0.001, *P* < 0.01 respectively) (Figure 2). There was no significant relationship between extracellular vesicles, the blast percentage, and WBC count.

*AML-EVS protected U937 cells from the cytotoxic effects of the chemotherapeutic agent*

**Figure 3:**
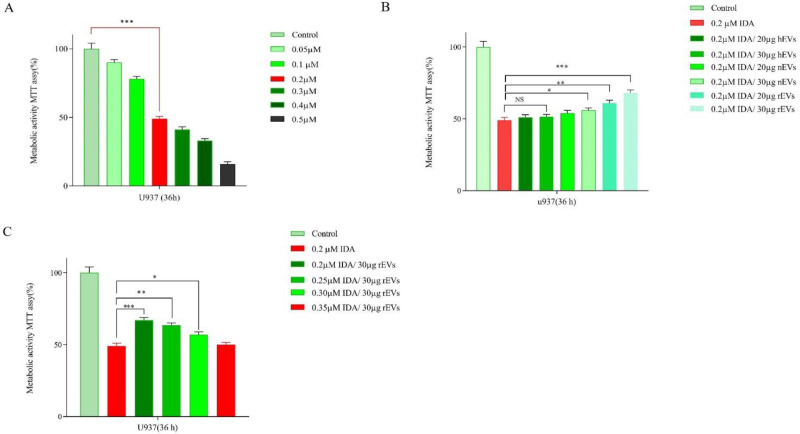
(A) Effects of IDA (0.05-0.5 μM) on the viability of U937 cells. The growth suppressive effect of IDA on U937 cells was assessed using MTT assay. IC_50_ pharmaceutical dose for U937 cells was 0.2 µM. (B) Data indicated that the anti-proliferative effects of IDA was attenuated in the presence of different AML-derived EVs. (C) When U937 cells were treated with relapsed AML-EVs (30 µg) in combination with IDA, there was a more significant elevation in the IC_50 _value of IDA. Data are mean ± SE of three independent experiments. Statistical significance was defined at ^*^P < 0.05, ^**^P < 0.01 and ^***^P < 0.001 compared to corresponding control

The cytotoxic effects of IDA, either alone or in combination with different concentrations of EVs, were evaluated in U937 cells. After treatment with different concentrations of IDA for 36 h, growth-suppressive effects were assessed by MTT assay. Our results showed that IDA inhibited cell proliferation of U937 cells with the IC_50_ values of 0.2 μM (Figure 3). U937 cells were then exposed to serial concentrations of EVs and the results of the MTT assay demonstrated that while EVs at the concentrations of 20 µg and 30 µg had the maximum proliferation effect on the cells, the higher concentrations of EVs induced apoptosis and halted cells proliferation. Based on these findings, these concentrations of EVs were selected for investigation in combination with different doses of IDA. Moreover, to examine whether EVs had the potential to induce IDA-resistance in U937 cells, we treated the cells with IDA, with the half-maximal (50%) inhibitory concentrations, and EVs at the concentrations of 20 and 30 µg. The obtained results demonstrated that the IC_50_ values of IDA in the presence of both new cases AML-EVs and relapsed AML-EVs increased from 0.2 μM to 0.35 μM in U937 cells, indicating that the apoptotic effect of IDA was attenuated in presence of EVs. 

*Induction of apoptosis in U937 cells after treatment with EVs*

**Figure 4:**
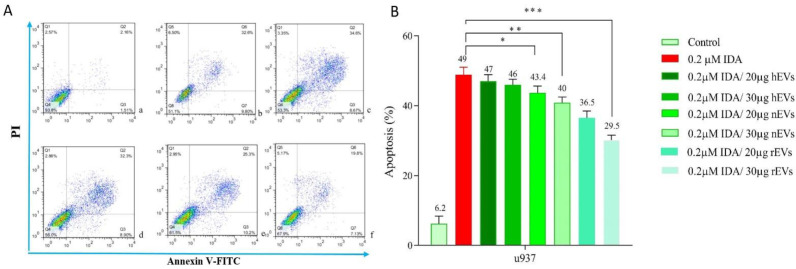
Effects of IDA on the induction of apoptosis in U937 cells. The cells were treated with 0.2 μM IDA and 20 and 30 µg of healthy, new case AML and relapsed AML-EVs respectively. Data are mean ± SE of three independent experiments. Statistical significance were defined at ^*^P < 0.05, ^**^P < 0.01 and ^***^P < 0.001compared to corresponding control

**Figure 5:**
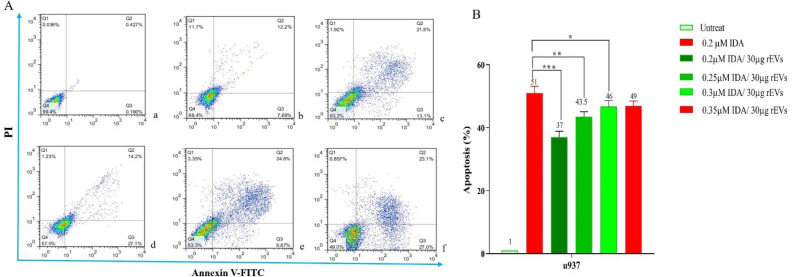
The result of annexin V/PI staining in EVs - or IDA+ 30 µg relapsed AML-EVs‐treated U937 during co-culture. Cells were treated for 36 h with either EVs (30 µg) or IDA+ EVs (30 µg). The relapsed AML-EVs protected the cells from IDA-induced apoptosis and enhanced the IDA IC_50_ in U937 cells (*P* < 0.001).

To determine the effect of EVs on IDA-induced apoptosis in U937 cells, we treated the cells with different concentrations of IDA, either alone or in combination with EVs, and then the cells were subjected to Annexin V-FITC and PI staining. In agreement with the results of the MTT assay, we observed that the exposure to AML- derived EVs significantly reduced the apoptotic effects of IDA as compared to the single-agent of IDA or IDA-healthy-EVs combination. Our data also underscored a higher induction of chemo-resistance for EVs isolated from relapsed patients than those isolated from new case-patients (Figures 4 and 5).

*MDR-1 and MRP1 expression in AML patients and EVs derived*

**Figure 6:**
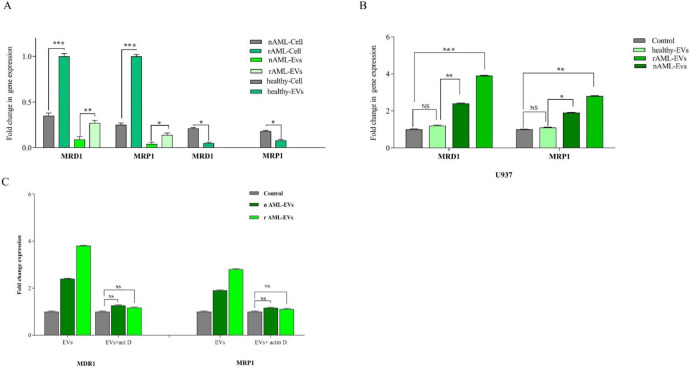
(A) MDR-1 and *MRP1* expression in healthy, new case AML and relapsed AML-cells and correspond EVs. We first analyzed the normalized expression of *MRD1* and *MRP1* in cellular and vesicular compartment of all subjects. The resulting data showed that the expression of these genes were lower in EVs as compared with AML parent cells. In new cases and relapsed patients, the fold changes of both genes in EVs were increased. (B) The expression of *MRD1* and *MRP1*in U937 cell treated with new cases and relapsed EVs were significantly increased. Data are mean ± SE of three independent experiments. (C) U937 cells was treated with actinomycin D (1 mg/mL) prior to EV incubation. After 24 h of EV integration, we did not observe any significant change in *MDR-1* and *MRP1* expression, indicating that the increase of these genes was not due to RNA transfer. Statistical significance were defined at ^*^P < 0.05, ^**^P < 0.01 and ^***^P < 0.001compared to corresponding control

To verify the presence of MDR-1 and *MRP1* in all three groups (healthy control, new case AML, and relapsed AML) and to correspond EVs, the expression of these genes was evaluated by qRT-PCR. Results demonstrated that EVs expressed *MDR-1* and *MRP1* in parallel with corresponding cells. Moreover, the higher levels of *MDR-1* and *MRP1* mRNA expression were detected in relapsed AML as compared to new cases AML (*P < .001*, *P* < .001 for *MRD1* and *P* < .001, *P < .05 *for *MRP1* in cell and EVs respectively) (Figure 6). 

*EVs-treated cells increased the expression of drug export pumps in U937 cells*

To identify what processes, at the molecular level, explain the reduction of IDA- induced apoptosis in U937 cells, we evaluate the expression of two important drug efflux pumps, *MRD1*, *MRP1*, in leukemic cells upon exposure to the single concentration of healthy-EVs, new case AML-Evs, and relapsed AML-EVs (30 µg/mL). Our results showed that the treatment of the cells with new case AML-EVs and relapsed AML-EVs increased the mRNA expression level of *MRD1* and *MRP1* as compared to the cells, which were treated with healthy EVs (Figure 6). As shown in Figure 6, the expression of *MRD1* and *MRP1* were increased by 2.4- and 3.8-fold changes in the presence of 30 µg new case AML-EVs and relapsed AML-EVs, respectively (*P* < .01, *P* < .001). *MRP1* expression was increased by 1.9- and 2.8-fold changes in 30 µg new case AML-EVs and relapsed AML-EVs compared to control, respectively (*P* < .05, *P* < .01). 

*The effects of EV on gene expression were due to de novo cell regulation rather than RNA transfer*

In order to investigate whether the effect of EVs on gene expression was due to the direct transfer of multidrug resistance mRNA or by the induction of gene expression in the recipient cells, U937 cells were treated with actinomycin D (1 µg/mL) prior to EV incubation. After 36 h of EV integration, expression of *MRD1* and *MRP1* were assessed. The resulting data showed that the expression of these genes was comparable prior and post EVs treatment, indicating that the elevation of these genes in the U937 cells untreated with actinomycin D was independent of direct RNA transfer by EVs (Figure 6). 

*AML-derived EVs decreased ROS level in U937 cells*

**Figure 7:**
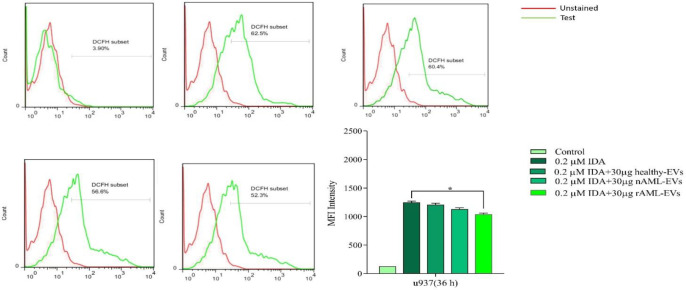
U937 cells were treated with or without 30 µg of healthy, new case AML and relapsed AML-EVs, and assayed for ROS production by using the fluorescent dye DCFDA. Representative histograms showed ROS generation in each experimental condition. Graph represented the mean values ± SE of the MFI of DCF. Statistical significance were defined at ^*^P < 0.05

To assess the effect of AML-derived EVs on the production of ROS in U937 cells, the cells were treated with IDA, either alone or combined with healthy-EVs, new case AML-EVs, and relapsed AML-EVs. Then, the production of ROS was evaluated using flow cytometry analysis. As shown in Figure 7, ROS generation was significantly reduced when IDA was used in combination with 30 µg relapsed AML-EVs as compared with those cells, which were only treated with IDA. Although the amount of ROS also decreased in the cells, which were treated with IDA in combination with new case AML-EVs and healthy-EVs, this reduction was not statistically significant. 

## Discussion

One of the main obstacles in the way to successful treatment of leukemia is drug-resistance, which is mediated through several mechanisms. Dysregulation in the expression level of both oncogenes and tumor suppressors, alteration in apoptotic pathways, as well as disruption in DNA repair responses are the most important mechanisms that are participated in decreasing the sensitivity of cancer cells to the conventional treatment strategies (21). To date, extracellular vesicles (EVs) has attracted tremendous attentions in cancer management, as they could participate in all aspects of formation and progression of malignant cells, such as differentiation, proliferation, migration, invasion, apoptosis and particularly chemo-resistance (22). It has been indicated that EVs could confer drug-resistance in cancer cells via vascular remodeling, transferring the regulatory apoptotic-related proteins, induction of oncogenic signaling axis, and over-activation of the drug efflux pumps (22). In this study, we aimed to investigate the influence of EVs on the induction of drug resistance in AML cells with a special focus on the role of drug efflux pumps and ROS production (23-25). 

In agreement with the previous studies, which reported the high concentrations of EVs in peripheral blood of numerous malignancies, we found that the protein content of AML patients at diagnosis and relapse was significantly higher as compared to healthy controls. In consistent, Caivano*.* Previously reported that the amount of EVs was elevated in various hematologic malignancies (26). In addition, Szczepanski and colleagues have been reported that the sera of AML patients had significantly greater protein content than those isolated from sera of normal controls (27). Hong also showed the protein levels of exosome fractions in plasma of refractory/relapsed AML patients was higher than healthy controls, which could be due to the responses to chemotherapy (28, 29). These findings suggested that there is a probable connection between EVs secretion and the proliferative capacity of the leukemic cells. 

AML-derived EVs could attenuate the effectiveness of different chemotherapy agents likely through induction of chemo-resistance in the recipient cells (23). In this way, we found that EVs obtained from the plasma of AML patients could confer resistance to the IDA in U937 cell line, as revealed by the significant elevation in the IC_50_ value of IDA. In parallel with this finding, Viola had reported AML-BMSC exosomes significantly protected MOLM-14 cells against the anti-leukemic effects of cytarabine and FLT3 inhibitor (30). Moreover, in another study, Patel demonstrated that upon exposure of pancreatic cancer cell lines to exosome, the IC_50 _value of gemcitabine increased in the cells as compared to the naive cells (31). 

In both malign and benign conditions, EVs, either arbitrary or specifically, loads diverse molecules from originated cells (32). It has been reported that when the proportion of cargo is greater in EVs than the corresponding cells, it is indicative of the specifically loading (30). The resulting data showed these molecules are loaded less on EVs than on parents’ cells that indicated probably other mechanisms, including miRNA transmission, could be involved in the occurrence of chemoresistance (12). However, further studies are needed in these patients to better understand the precise mechanisms involved in the occurrence of resistance. The ABC transporters, which express ubiquitously on normal tissues, have been identified as drug-resistance proteins (17, 33 and 34). Upregulation of *MRD1* and *MRP1* was also reported in human osteosarcoma (18). Consistently, the results of the present study declared that the expression level of both *MRD1* and *MRP1* were higher in relapsed AML patients as compared to the new cases. Over-activation of the ABC efflux pumps is a common occurrence in AML and the expression of these resistance genes are proposed as independent predictors for treatment outcome in adults (35). Here, we showed that in agreement with *MDR1*, the expression of *MRP1* was increased in EVs derived from AML patients as compared with healthy controls. Studies of EVs isolated from tumor-cell supernatants indicated that EVs are enriched in the specific proteins, lipids, and nucleic acids, which were presented in the parental cell, while some other contents are not presented in them, reflecting the fact that EVs biogenesis is not a random process (36). Moreover, bioanalyzer spectrum of bone marrow stromal cells (BMSC)-derived exosome RNA showed a relatively greater abundance of small RNAs as compared to the parent cell (30). It has been reported that EVs could induce chemo-resistance in tumor cells through carrying MDR-associated proteins and the ABC transporter efflux system (37). It has been shown that docetaxel-resistant prostate cancer and cisplatin-resistant ovarian cancer cells released more exosomes than sensitive cells (38). In our study, *MDR1* and *MRP1* expressions were increased in U937 cells, which were treated with new case AML-EVs and relapsed AML-EVs. The association between EVs and induction of chemo-resistance could be regulated through different signaling pathways, such as MAPK/ERK, CaM-Ks/Raf/MEK/ERK, and PI3K (39-41).

We also examined whether the overexpression of drug efflux pumps in U937 cells was mediated by a direct transfer of *MRD1* and *MRP1* mRNAs or was due to the induction of gene expression. In this regard, the de novo mRNA expression of U937 cells was halted using actinomycin D treatment just a few hours before treatment with EVs. Of note, the gene expression of *MRD1* and *MRP1* did not change significantly in the presence of actinomycin D, suggesting that the de novo mechanisms were involved in the elevation of ABC efflux pumps upon exposure to the malignant EVs. In parallel with our experiments, Crompot has reported the active uptake of the BM-MSC EVs by chronic lymphocytic leukemia (CLL) cells could modify the gene expression even after exposure to actinomycin D, which abolished the gene expression system in CLL cells (24). On the contrary, the serial analysis clearly showed that EVs mediate a horizontal transfer of genetic information to recipient cells (12, 23 and 42). 

Under normal conditions, ROS production and ROS elimination are balanced; however, it has also been reported that the production of ROS could be an adaptive response to age, sex, tumor growth or stress conditions during the cell´s lifespan (43). EVs and ROS are closely interrelated, as EVs can produce or detoxify ROS, and on the other hand, ROS could also increase EVs production. Reciprocal effects depend on both the conditions of the EVs correspond to cells, as well as the environmental conditions (44). It is well-established that any aberrancies in the redox system due to the fluctuation in ROS levels could reduce the sensitivity of cancer cells to the chemotherapeutic drugs (45). Our results showed that when U937 cells were treated with AML-derived EVs, there was a reduction in the intracellular level of ROS. This inhibition was even more significant in those cells, which were exposed to relapsed ALM-EVs, which was in agreement with the results of both viability and apoptosis assays, reflecting the fact that malignant EVs could bypass the cytotoxic effect of IDA and increase the survival of the cells. In parallel with our research, F Alcayaga-Miranda indicated that MSCs secreted exosomes reduced ROS in prostate PC3 tumor cells (46). Jafarzadeh N also represented that K562-derived exosomes dose-dependently reduced the intracellular level of ROS in bone marrow mesenchyme stem cells (BM-MSCs) (47). On this basis, GK Patel suggested that promoting ROS detoxification could confer chemo-resistance in pancreatic cancer (31). Although numerous studies were suggesting that reduction in ROS production could mediate chemo-resistance in cancer cells, there are some conflicting results. S Dutta has shown that the interactions and the uptake of breast cancer cell lines-derived exosome by HMECs serve as a signal to induce ROS in the mammary epithelial cells, suggesting that probably the behavior of the cells and their responses could be varied due to EVs cargo content (48). 

**Table 1:** Characteristics of AML patients included in this study

**Newly diagnosed AML patient (n = 7)**	
Median Age (years)	56 (Range, 33–74)
**FAB classification**	
M1 M2	2 5
Blast present (%)	47 (Range, 29-63)
WBC count (µL)	17300 × 10^3 ^(4600-31200)
**Relapsed diagnosed AML patient (n = 4)**	
Median Age (years)	44 (Range, 9–74)
**FAB classification**	
M1 M2	1 3
Blast present (%)	31 (Range, 14-58)
WBC count (µL)	10700 × 10^3 ^(5100-18100)
**Healthy subjects (n = 10)**	
Median Age (years)	49 (Range 17-69)
WBC count (µL)	5100 × 10^3^(3800-8600)

**Table 2:** Primer sequences of the endogenous control and drug resistant genes

**Gene**	**Primer sequence (5′ to 3′)**	**Primer length (bp)**	**Amplicon length (bp)**
*ABL*	F: AGTCTCAGGATGCAGGTGCT R: TAGGCTGGGGCTTTTTGTAA	20	124
		20	
*MRD1(ABCB1)*	F:GAGGCCGCTGTTCGTTTCCT TTAGGTC R: AGATTCATTCCGACCTCGCGCTCCT	26	102
		25	
*MRP1(ABCC1)*	F: CGGATGTCATCTGAAATGGGA R: GAGCTGTCTCCTGGATTTGC	21	103
		20	

## Conclusion

Arising resistant diseases is of an utmost important issue in medicine, particularly in the path of cancer treatment. Our data revealed that EVs could confer IDA resistance in a sensitive AML-derived cell line. With more depth analyses, we found that resistant phenotype was mediated, at least in part, through induction of *MRD1* and *MRP1* gene expression, but not a direct transfer of these genes. We also found that ROS reduction may be another mechanism, which could result in the generation of resistant cells upon exposure to relapsed AML-derived EVs. A better understanding of other plausible mechanisms involved in arising of resistant phenotype may lead to the development of more effective treatment for AML patients in the future. 

## References

[1] Roug AS, Ommen HB (2019). Clinical use of measurable residual disease in acute myeloid leukemia. Curr. Treat. Options Oncol..

[2] Saito Y, Kitamura H, Hijikata A, Tomizawa-Murasawa M, Tanaka S, Takagi S, Uchida N, Suzuki N, Sone A, Najima Y (2010). Identification of therapeutic targets for quiescent, chemotherapy-resistant human leukemia stem cells. Sci. Transl. Med..

[3] Rowe JM, Kim HT, Cassileth PA, Lazarus HM, Litzow MR, Wiernik PH, Tallman MS (2010). Adult patients with acute myeloid leukemia who achieve complete remission after 1 or 2 cycles of induction have a similar prognosis: a report on 1980 patients registered to 6 studies conducted by the Eastern Cooperative Oncology Group. Cancer.

[4] Behrmann L, Wellbrock J, Fiedler W (2018). Acute myeloid leukemia and the bone marrow niche—take a closer look. Front. Oncol..

[5] Landau DA, Carter SL, Getz G, Wu CJ (2014). Clonal evolution in hematological malignancies and therapeutic implications. Leukemia.

[6] Vosberg S, Greif PA (2019). Clonal evolution of acute myeloid leukemia from diagnosis to relapse. Genes Chromosom. Cancer.

[7] Shlush LI, Mitchell A, Heisler L, Abelson S, Ng SW, Trotman-Grant A, Medeiros JJ, Rao-Bhatia A, Jaciw-Zurakowsky I, Marke R (2017). Tracing the origins of relapse in acute myeloid leukaemia to stem cells. Nature.

[8] Revenfeld ALS, Bæk R, Nielsen MH, Stensballe A, Varming K, Jørgensen M (2014). Diagnostic and prognostic potential of extracellular vesicles in peripheral blood. Clin. Ther..

[9] Van Niel G, d’Angelo G, Raposo G (2018). Shedding light on the cell biology of extracellular vesicles. Nat. Rev. Mol. Cell Biol..

[10] Xu R, Rai A, Chen M, Suwakulsiri W, Greening DW, Simpson RJ (2018). Extracellular vesicles in cancer—implications for future improvements in cancer care. Nat. Rev. Clin. Oncol..

[11] Jabalee J, Towle R, Garnis C (2018). The role of extracellular vesicles in cancer: cargo, function and therapeutic implications. Cells.

[12] Bouvy C, Wannez A, Laloy J, Chatelain C, Dogné JM (2017). Transfer of multidrug resistance among acute myeloid leukemia cells via extracellular vesicles and their microRNA cargo. Leukemia Res..

[13] Milman N, Ginini L, Gil Z (2019). Exosomes and their role in tumorigenesis and anticancer drug resistance. Drug Resist. Updat..

[14] Locher KP (2016). Mechanistic diversity in ATP-binding cassette (ABC) transporters. Nat. Struct. Mol. Biol..

[15] Gottesman MM, Fojo T, Bates SE (2002). Multidrug resistance in cancer: role of ATP–dependent transporters. Nat. Rev. Cancer.

[16] Goler-Baron V, Assaraf YG (2011). Structure and function of ABCG2-rich extracellular vesicles mediating multidrug resistance. PLoS One.

[17] Borst P, Elferink RO (2002). Mammalian ABC transporters in health and disease. Annu. Rev. Biochem..

[18] Torreggiani E, Roncuzzi L, Perut F, Zini N, Baldini N (2016). Multimodal transfer of MDR by exosomes in human osteosarcoma. Int. J. Oncol..

[19] Wang X, Qiao D, Chen L, Xu M, Chen S, Huang L, Wang F, Chen Z, Cai J, Fu L (2019). Chemotherapeutic drugs stimulate the release and recycling of extracellular vesicles to assist cancer cells in developing an urgent chemoresistance. Mol. Cancer.

[20] Pospichalova V, Svoboda J, Dave Z, Kotrbova A, Kaiser K, Klemova D, Ilkovics L, Hampl A, Crha I, Jandakova E (2015). Simplified protocol for flow cytometry analysis of fluorescently labeled exosomes and microvesicles using dedicated flow cytometer. J. Extracell. Vesicles.

[21] Rebucci M, Michiels C (2013). Molecular aspects of cancer cell resistance to chemotherapy. Biochem. Pharmacol..

[22] Yang C, Yang H, Liu J, Zhu L, Yu S, Zhang X, Gao L (2019). Focus on exosomes: Novel pathogenic components of leukemia. Am. J. Cancer Res..

[23] Mc Namee N, O’Driscoll L (2018). Extracellular vesicles and anti-cancer drug resistance. Biochim. Biophys. Acta Rev. Cancer.

[24] Crompot E, Van Damme M, Pieters K, Vermeersch M, Perez-Morga D, Mineur P, Maerevoet M, Meuleman N, Bron D, Lagneaux L (2017). Extracellular vesicles of bone marrow stromal cells rescue chronic lymphocytic leukemia B cells from apoptosis, enhance their migration and induce gene expression modifications. Haematologica.

[25] Camussi G, Deregibus MC, Bruno S, Cantaluppi V, Biancone L (2010). Exosomes/microvesicles as a mechanism of cell-to-cell communication. Kidney Int..

[26] Caivano A, Laurenzana I, De Luca L, La Rocca F, Simeon V, Trino S, D’Auria F, Traficante A, Maietti M, Izzo T (2015). High serum levels of extracellular vesicles expressing malignancy-related markers are released in patients with various types of hematological neoplastic disorders. Tumor Biol..

[27] Szczepanski MJ, Szajnik M, Welsh A, Whiteside TL, Boyiadzis M (2011). Blast-derived microvesicles in sera from patients with acute myeloid leukemia suppress natural killer cell function via membrane-associated transforming growth factor-β1. Haematologica.

[28] Hong CS, Sharma P, Yerneni SS, Simms P, Jackson EK, Whiteside TL, Boyiadzis M (2017). Circulating exosomes carrying an immunosuppressive cargo interfere with cellular immunotherapy in acute myeloid leukemia. Sci. Rep..

[29] Hong CS, Muller L, Whiteside TL, Boyiadzis M (2014). Plasma exosomes as markers of therapeutic response in patients with acute myeloid leukemia. Front. Immunol..

[30] Viola S, Traer E, Huan J, Hornick NI, Tyner JW, Agarwal A, Loriaux M, Johnstone B, Kurre P Alterations in acute myeloid leukaemia bone marrow stromal cell exosome content coincide with gains in tyrosine kinase inhibitor resistance. Br. J. Haematol..

[31] Patel GK, Khan MA, Bhardwaj A, Srivastava SK, Zubair H, Patton MC, Singh S, Singh AP (2017). Exosomes confer chemoresistance to pancreatic cancer cells by promoting ROS detoxification and miR-155-mediated suppression of key gemcitabine-metabolising enzyme, DCK. Br. J. Cancer.

[32] Sato S, Weaver AM (2018). Extracellular vesicles: important collaborators in cancer progression. Essays Biochem..

[33] Borst P, Evers R, Kool M, Wijnholds J (2000). A family of drug transporters: the multidrug resistance-associated proteins. J. Natl. Cancer Inst..

[34] Ambudkar SV, Dey S, Hrycyna CA, Ramachandra M, Pastan I, Gottesman MM (1999). Biochemical, cellular, and pharmacological aspects of the multidrug transporter. Annu. Rev. Pharmacol. Toxicol..

[35] Schaich M, Soucek S, Thiede C, Ehninger G, Illmer T, group SAs (2005). MDR1 and MRP1 gene expression are independent predictors for treatment outcome in adult acute myeloid leukaemia. Br. J. Haematol..

[36] Ohno SI, Ishikawa A, Kuroda M (2013). Roles of exosomes and microvesicles in disease pathogenesis. Adv. Drug Deliv. Rev..

[37] Jones P, George A (2004). The ABC transporter structure and mechanism: perspectives on recent research. Cell. Mol. Life Sci..

[38] Yousafzai NA, Wang H, Wang Z, Zhu Y, Zhu L, Jin H, Wang X (2018). Exosome mediated multidrug resistance in cancer. Am. J. Cancer Res..

[39] Ning K, Wang T, Sun X, Zhang P, Chen Y, Jin J, Hua D (2017). UCH-L1-containing exosomes mediate chemotherapeutic resistance transfer in breast cancer. J. Surg. Oncol..

[40] Liu J, Zhang Y, Liu A, Wang J, Li L, Chen X, Gao X, Xue Y, Zhang X, Liu Y (2016). Distinct dasatinib-induced mechanisms of apoptotic response and exosome release in imatinib-resistant human chronic myeloid leukemia cells. Int. J. Mol. Sci..

[41] Ji R, Zhang B, Zhang X, Xue J, Yuan X, Yan Y, Wang M, Zhu W, Qian H, Xu W (2015). Exosomes derived from human mesenchymal stem cells confer drug resistance in gastric cancer. Cell Cycle.

[42] Boyiadzis M, Hong CS, Whiteside TL (2016). Circulating exosomes carrying an immunosuppressive cargo interfere with adoptive cell therapy in acute myeloid leukemia. Blood.

[43] Bodega G, Alique M, Puebla L, Carracedo J, Ramírez R (2019). Microvesicles: ROS scavengers and ROS producers. J. Extracell. Vesicles.

[44] Benedikter BJ, Weseler AR, Wouters EF, Savelkoul PH, Rohde GG, Stassen FR (2018). Redox-dependent thiol modifications: implications for the release of extracellular vesicles. Cell. Mol. Life Sci..

[45] Kim EK, Jang M, Song MJ, Kim D, Kim Y, Jang HH (2019). Redox-mediated mechanism of chemoresistance in cancer cells. Antioxidants.

[46] Alcayaga-Miranda F, González PL, Lopez-Verrilli A, Varas-Godoy M, Aguila-Díaz C, Contreras L, Khoury M (2016). Prostate tumor-induced angiogenesis is blocked by exosomes derived from menstrual stem cells through the inhibition of reactive oxygen species. Oncotarget.

[47] Jafarzadeh N, Safari Z, Pornour M, Amirizadeh N, Forouzandeh Moghadam M, Sadeghizadeh M (2019). Alteration of cellular and immune-related properties of bone marrow mesenchymal stem cells and macrophages by K562 chronic myeloid leukemia cell derived exosomes. J. Cell. Physiol..

[48] Dutta S, Warshall C, Bandyopadhyay C, Dutta D, Chandran B (2014). Interactions between exosomes from breast cancer cells and primary mammary epithelial cells leads to generation of reactive oxygen species which induce DNA damage response, stabilization of p53 and autophagy in epithelial cells. PLoS One.

